# Predictors of advanced fibrosis in elderly patients with biopsy-confirmed nonalcoholic fatty liver disease: the GOASIA study

**DOI:** 10.1186/s12876-020-01240-z

**Published:** 2020-04-06

**Authors:** Panyavee Pitisuttithum, Wah Kheong Chan, Panida Piyachaturawat, Kento Imajo, Atsushi Nakajima, Yosuke Seki, Kazunori Kasama, Satoru Kakizaki, Jian Gao Fan, Myeong Jun Song, Seung Kew Yoon, Yock Young Dan, Laurentius Lesmana, Khek Yu Ho, Khean Lee Goh, Vincent Wai Sun Wong, Sombat Treeprasertsuk

**Affiliations:** 1 Department of Medicine, Chulalongkorn University, Bangkok, Thailand; 2 Department of Medicine, University of Malaya, Kuala Lumpur, Malaysia; 3 Department of Gastroenterology and Hepatology, Yokohama City University Graduate School of Medicine, Yokohama, Japan; 4 Weight Loss and Metabolic Surgery Centre, Yotsuya Medical Cube, Tokyo, Japan; 5 Department of Gastroenterology and Hepatology, Gunma University Graduate School of Medicine, Gunma, Japan; 6 Department of Gastroenterology, Shanghai Jiaotong University School of Medicine, Shanghai, China; 7 Department of Internal Medicine, The Catholic University of Korea, Seoul, Korea; 8 Department of Medicine, National University of Singapore, Singapore, Singapore; 9 Digestive Disease and Oncology GI Centre, Medistra Hospital, Jakarta, Indonesia; 10 Department of Medicine and Therapeutics, The Chinese University of Hong Kong, Hong Kong, China

**Keywords:** Advanced fibrosis, Elderly patients, Nonalcoholic fatty liver disease (NAFLD)

## Abstract

**Background:**

The Gut and Obesity in Asia (GOASIA) Workgroup was formed to study obesity and gastrointestinal diseases in the Asia Pacific region. We aimed to 1) compare the characteristics of elderly (i.e. age ≥ 60) vs. non-elderly patients with biopsy-proven nonalcoholic fatty liver disease (NAFLD); 2) identify predictors of advanced fibrosis in elderly patients with NAFLD; and 3) assess the performance of non-invasive fibrosis scores in the prediction of advance fibrosis in the elderly population.

**Methods:**

We abstracted the data of 1008 patients with NAFLD from nine centers across eight countries. Characteristics of elderly and non-elderly patients with NAFLD were compared using 1:3 sex-matched analysis.

**Results:**

Of the 1008 patients, 175 were elderly [age 64 (62–67) years], who were matched with 525 non-elderly patients [46 (36–54) years]. Elderly patients were more likely to have advanced fibrosis (35.4% vs. 13.3%; *p* < 0.001). By multivariable analysis, factors associated with advanced fibrosis in elderly patients included female sex [odds ratio (OR) 3.21; 95% confidence interval (CI) 1.37–7.54] and hypertension (OR 3.68; 95%CI 1.11–12.23). The area under receiver-operating characteristics curve (95% CI) of aspartate aminotransferase-to-platelet ratio index, NAFLD fibrosis score and Fibrosis-4 index for predicting advanced fibrosis in elderly patients were 0.62 (0.52–0.72), 0.65 (0.55–0.75) and 0.64 (0.54–0.74) respectively.

**Conclusions:**

Elderly patients with NAFLD had a higher prevalence of advanced fibrosis than non-elderly patients. Female and hypertension were predicting factors for advanced fibrosis in the elderly. Non-invasive fibrosis scores had a lower specificity in elderly.

## Background

Nowadays, nonalcoholic fatty liver disease (NAFLD) is a growing problem and is becoming a major cause of chronic liver disease worldwide. NAFLD broadly encompasses two conditions with different prognosis: nonalcoholic fatty liver (NAFL) and nonalcoholic steatohepatitis (NASH). The prevalence of NASH has consistently been reported to increase with age besides being associated with obesity, diabetes mellitus and the metabolic syndrome [1–3]. Due to population aging, NAFLD is projected to increase globally.

Recently, Ballestri and colleagues conducted a comprehensive review of NAFLD studies and found age, sex and the metabolic syndrome to be major factors influencing NAFLD onset and progression. The incidence of NAFLD is higher in men [4, 5]. In men, the prevalence of NAFLD increases during young adulthood to middle age and decline after the age of 50–60 years [6, 7]. In contrast, NAFLD is more common among elderly women with metabolic comorbidities [8]. In premenopausal women, the incidence of NAFLD is low but increases rapidly after menopause [9, 10]. The striking gender difference has long been reported in NAFLD.

The global prevalence of NAFLD is about 25% [11], while in Asia the disease has been reported to affect up to 30% of the general population in some studies [12–14]. However, data in the elderly population are limited. Currently, the proportion of older population is growing dramatically due to increased life expectancy and declining birth rates. Accordingly, the number of NAFLD patients would also inevitably increase. Predicting advanced fibrosis is necessary because of the higher risk of complications from chronic liver disease such as cirrhosis and hepatocellular carcinoma. In order to evaluate the liver disease severity, liver biopsy is gold standard. However, it is an invasive procedure and may be limited by sampling variability. So far there is no specific recommendation for elderly NAFLD in terms of diagnostic and fibrosis assessment [15, 16]. Previous studies have shown that non-invasive fibrosis scores, including fibrosis-4 (FIB-4) and NAFLD fibrosis score (NFS) have low specificity in elderly NAFLD patients [17].

The Gut and Obesity in Asia (GO ASIA) Workgroup was formed in November 2014 with the aim of studying relationship between obesity and gastrointestinal and liver diseases in the Asia Pacific region through multi-national collaborative studies [18, 19]. A database consisting of biopsy-proven NAFLD patients was formed through contribution from participating members of the GO ASIA Workgroup. We aimed to investigate factors associated with liver fibrosis in elderly (i.e. age ≥ 60) vs. non-elderly patients with biopsy-proven NAFLD, and to identify predictors of advanced fibrosis in elderly patients. In addition, we evaluated the performance of non-invasive fibrosis scores in elderly NAFLD patients.

## Methods

### Participating centers and cases

This is a cross-sectional study of 1008 patients with biopsy-proven NAFLD in nine centers across eight countries in the GO ASIA Workgroup [19]. The centers that participated in this study and the duration of data collection were listed as follows: The Chinese University of Hong Kong, Hong Kong collected data from September 2006 to October 2015; University of Malaya, Malaysia collected data from November 2012 to October 2015; Chulalongkorn University, Thailand collected data from January 2007 to February 2014; Yotsuya Medical Cube / Gunma University Graduate School of Medicine, and Yokohama City University Graduate School of Medicine, Japan collected data from October 2009 to February 2016; Shanghai Jiaotong University School of Medicine, China collected data from June 2012 to December 2013; The Catholic University of Korea, Korea collected data from January 2009 to February 2016; National University of Singapore, Singapore collected data from October 2002 to October 2015; and Digestive Disease and Oncology GI Centre, Medistra Hospital, Indonesia collected data from May 2011 to September 2015. Ethical approval was obtained from each center prior to the commencement of the study. Verbal informed consent was obtained from all patients for being included in the study and the institutional review board approved the use of verbal consent.

NAFLD was diagnosed based on ultrasonography findings of fatty liver with exclusion of viral hepatitis B and C infection, significant alcohol intake, and the use of medications that can cause hepatic steatosis. Persistent elevated serum aminotransferase levels were the main indication for liver biopsy. Patients with other causes of chronic liver disease, incomplete histological data and without significant hepatic steatosis were excluded from the analyses. Demographic, anthropometric, clinical and laboratory data were collected using a standard protocol.

### Definitions

Elderly patients were defined as age ≥ 60 years. Obesity was defined as body mass index (BMI) ≥ 25.0 kg/m^2^ [20]. Central obesity was defined as waist circumference (WC) > 90 cm for men and > 80 cm for women [21]. A patient was considered to have diabetes mellitus if there was a self-reported history of diabetes mellitus, or if fasting blood sugar was ≥ 7.0 mmol/L. A patient was considered to have dyslipidemia if there was a self-reported history of dyslipidemia, if the serum total cholesterol (TC) was ≥ 5.2 mmol/L, if the serum triglyceride (TG) was ≥ 1.7 mmol/L, if the serum high-density lipoprotein (HDL) was < 1.0 mmol/L for men or < 1.3 mmol/L for women, or if the serum low-density lipoprotein (LDL) was ≥ 3.4 mmol/L. All laboratory parameters included in the study were within 3 months before or after liver biopsy obtained. Hypertension was based on self-reported history. If they were using any drugs as evidence of the corresponding metabolic disorders also considered as having diabetes mellitus, dyslipidemia or hypertension.

### Non-invasive assessment of advanced fibrosis in patients with NAFLD

The performance of the NAFLD fibrosis score (NFS), fibrosis 4 (FIB-4) score, and aspartate aminotransferase (AST) to platelet ratio index (APRI) in prediction of advanced fibrosis in the elderly and non-elderly NAFLD patients were assessed.

### Histological data

Histopathological findings were reported according to the Non-Alcoholic Steatohepatitis Clinical Research Network Scoring System [22]. NAFLD Activity Score (NAS) was defined as the un-weighted sum of the scores for steatosis which was the presence of significant hepatic steatosis (> 5% of hepatocytes) (0–3), lobular inflammation (0–3), and ballooning (0–2); thus ranging from 0 to 8. NASH was defined as steatosis with hepatocyte ballooning and inflammation with/without fibrosis [23].

The slides were review by pathologist in each center. Fibrosis stages 3 and 4 were considered as advanced fibrosis (F3–4).

### Statistical analysis

We performed 1:3 matching by sex for elderly and non-elderly NAFLD patients. Continuous variables were reported as mean, standard deviation and analyzed using t-test. For non-normal distribution data were reported as median, interquartile range and analyzed using Mann-Whitney U test. Categorical variables were reported as percentages and analyzed using chi-square test. Factors associated with advanced fibrosis in elderly group were identified using binary logistic regression analysis. Multivariable analysis was performed by including all variables that had *p*-values < 0.1 from univariable analysis. Area under receiver-operating characteristics curve (AUROC) and its 95% confidence intervals were generated to determine performance of fibrosis prediction scores. A two-tailed *P*-value < 0.05 was considered statistically significant. Analyses were performed using SPSS 22.0 (SPSS Inc., Chicago, Illinois, U.S.).

## Results

### Characteristics of elderly and non-elderly NAFLD patients

Of 1008 patients with biopsy-proven NAFLD, 175 patients were elderly, who were matched with 525 non-elderly patients. The percentages of elderly NAFLD patients from each country included in the study are shown in supplemental table 1. The median (IQR) age of elderly NAFLD and non-elderly NAFLD patients were 64 (62–67) and 46 (36–54) years. In the elderly group, 102 (58.3%) patients were female. Characteristics of elderly and non-elderly NAFLD patients in different centers are shown in supplemental table 2.

The elderly NAFLD patients were more likely to have diabetes mellitus, hypertension and dyslipidemia than non-elderly patients. Elderly NAFLD patients had significantly lower BMI [27.19 (24.73–30.34) vs 29.62 (26.45–35) kg/m^2^, *p* < 0.001] and hip circumference [97 (87.75–104) vs 100 (88–108) cm, *p* = 0.037]. Elderly NAFLD patients had lower alanine aminotransferase [55 (38–94) vs 61 (37–90) U/L, *p* = 0.002], platelet count [227.5 (177.5–280.75) vs 251 (202–297) × 10^9^/L, *p* < 0.001], white blood count [6.57 (5.6–8) vs 7.3 (5.9–8.8) × 10^3^/mm^3^, *p* = 0.015], total cholesterol [4.4 (3.5–5.33) vs 5 (4.3–5.7) mmol/L, *p* < 0.001] and LDL cholesterol [2.3 (1.79–3.25) vs 3 (2.43–3.7) mmol/L, p < 0.001] than non-elderly NAFLD patients. Elderly patients had significantly higher hemoglobin A1c than non-elderly patients [6.65 (5.9–7.33) vs 6.1 (5.6–7.2) %, *p* < 0.001] (Table 1).

**Table 1:** Characteristics of elderly and non-elderly NAFLD patients were compared using1:3 sex-matched analysis

Characteristics	Elderly NAFLD patients	Non-elderly NAFLD patients	***P*** value
**median (IQR); n (%)**	**(n = 175)**	**(*n*** **= 525)**	
**Age (year)**	64 (62–67)	46 (36–54)	**< 0.001**
**Female**	102 (58.3%)	306 (58.3%)	1
**Weight (kg)**	69.45 (62.08–78.13)	78.5 (68.1–97.3)	**< 0.001**
**Height (m)**	1.6 (1.54–1.67)	1.6 (1.6–1.7)	**< 0.001**
**BMI (kg/m**^**2**^**)**	27.19 (24.73–30.34)	29.62 (26.45–35)	**< 0.001**
**BMI ≥ 25 (kg/m**^**2**^)	126 (72%)	443 (84.4%)	**< 0.001**
**Waist circumference (cm)**	92.05 (81.75–99.63)	91.2 (78–102.5)	0.813
**Hip circumference (cm)**	97 (87.75–104)	100 (88–108)	**0.037**
**Central obesity**	126 (85.1%)	350 (85.6%)	0.987
**Diabetes**	122 (69.7%)	237 (45.1%)	**< 0.001**
**Hypertension**	122 (74.8%)	189 (43.8%)	**< 0.001**
**Dyslipidemia**	148 (84.6%)	374 (71.2%)	**< 0.001**
**Albumin (g/L)**	43 (41–46)	44 (41–46)	0.177
**ALT (U/L)**	55 (38–94)	61 (37–90)	**0.002**
**AST (U/L)**	43 (26–66)	40 (29.8–68)	0.327
**ALP (U/L)**	69 (53.25–91)	67 (48.5–91.5)	0.942
**GGT (U/L)**	53 (37–87)	50 (31–86)	0.198
**HbA1c (%)**	6.65 (5.9–7.33)	6.1 (5.6–7.2)	**< 0.001**
**Total cholesterol (mmol/L)**	4.4 (3.5–5.33)	5 (4.3–5.7)	**< 0.001**
**LDL cholesterol (mmol/L)**	2.3 (1.79–3.25)	3 (2.43–3.7)	**< 0.001**
**HDL cholesterol (mmol/L)**	1.25 (1.1–1.44)	1.2 (1.01–1.5)	0.248
**Triglycerides (mmol/L)**	1.4 (1.1–1.8)	1.4 (1.1–1.9)	0.509
**Hemoglobin (g/dL)**	14.1 (13.4–15.08)	13.9 (13.1–15.1)	0.775
**Platelet (10**^**9**^**/L)**	227.5 (177.5–280.75)	251 (202–297)	**< 0.001**
**WBC (10**^**9**^**/L)**	6.57 (5.6–8)	7.3 (5.9–8.8)	**0.015**
**NAFLD Activity Score (NAS)**	4 (3–5)	4 (3–5)	0.332
**NAS ≥ 5**	58 (33.1%)	221 (40.2%)	0.097
**NASH**	118 (67.4%)	335 (63.8%)	0.386
**Significant fibrosis (≥F2): histopathology**	86 (49.1%)	138 (26.3%)	**< 0.001**
**Advanced fibrosis (≥F3): histopathology**	62 (35.4%)	70 (13.3%)	**< 0.001**
**Cirrhosis**	27 (15.4%)	25 (4.8%)	**< 0.001**
**NAFLD fibrosis score (NFS)**^a^	−0.75 (1.49)	−1.91 (1.88)	**< 0.001**
**APRI score**^a^	1.18 (2.02)	0.74 (1.55)	**0.02**
**FIB-4 score**^a^	3.88 (6.72)	1.58 (3.93)	**< 0.001**

*APRI* AST to platelet ratio index, *BMI* body mass index; Non-Alcoholic Steatohepatitis (NAS) score, ^a^mean (SD)

**Fig. 1:**
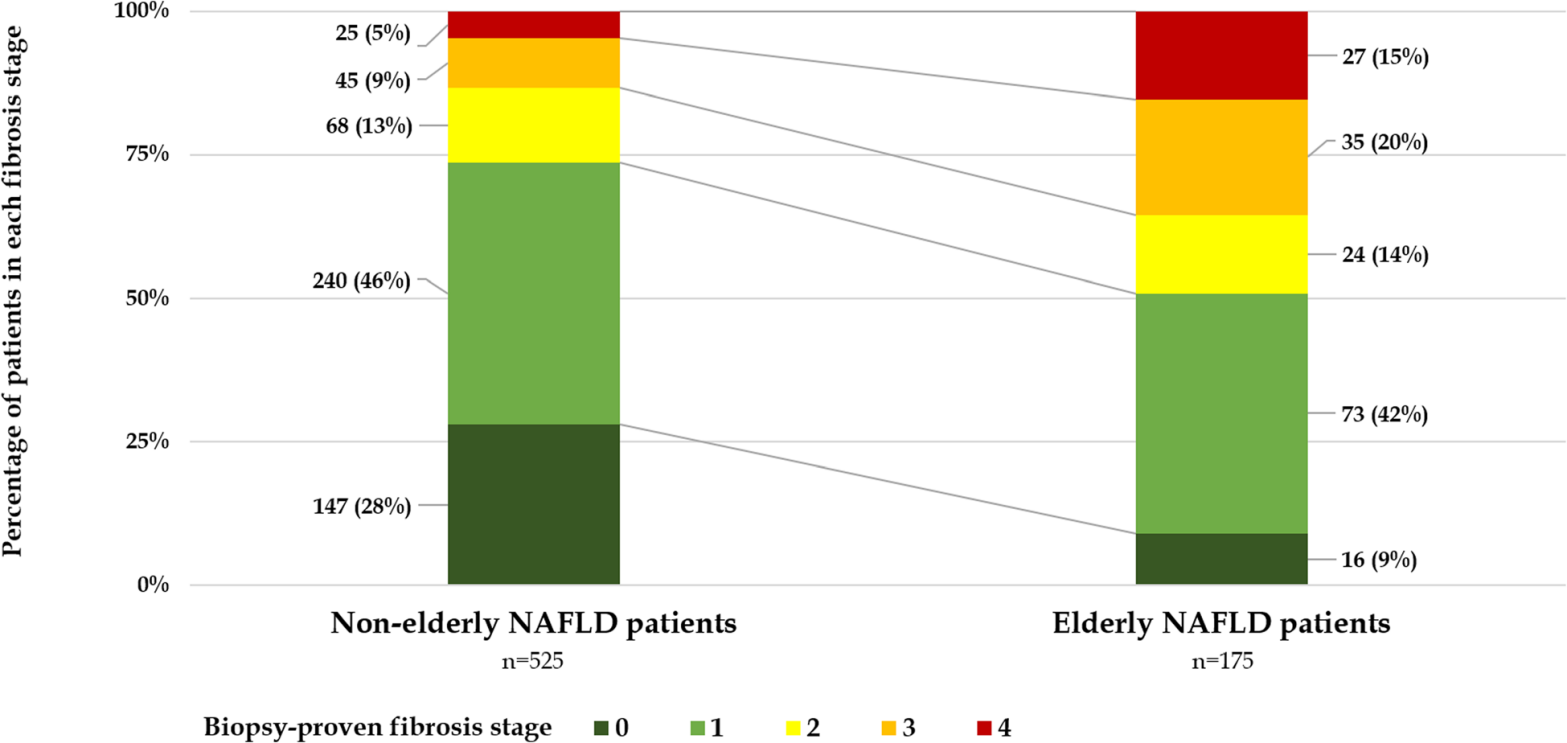
The percentages of fibrosis stages compared between elderly and non-elderly NAFLD patients: elderly NAFLD patients had significantly more patients with advanced fibrosis (F3–4; 35.4% vs. 13.4%; *P* < 0.01)

For the pathology findings, NASH was not difference between elderly and non-elderly NAFLD patients (67.4% vs.63.8%, *p* = 0.386). Elderly NAFLD patients were more likely to have advanced fibrosis (F3–4; 35.4% vs. 13.3%, *p* < 0.001) and cirrhosis (15.4% vs. 4.8%, p < 0.001) than non-elderly NAFLD patients (Fig. 1). Elderly patients also had higher fibrosis scores: APRI (1.18 ± 2.02 vs. 0.74 ± 1.55, *p* = 0.02), NFS (− 0.75 ± 1.49 vs. -1.91 ± 1.88, p < 0.001), and FIB-4 index (3.88 ± 6.72 vs. 1.58 ± 3.935, p < 0.001) (Table 1).

### Characteristics of elderly NAFLD patients with and without advanced fibrosis

Focusing on the elderly NAFLD patients, over one-third of them had advanced fibrosis (35.4%) including stage 3 fibrosis (F3; 20%) and stage 4 fibrosis (F4; 15.4%). Elderly NAFLD patients with advanced fibrosis were more likely to be female (72.6% vs. 50.4%; *p* = 0.005), obesity (83.9% vs 65.5%; *p* = 0.01), central obesity (94.7% vs. 79.1%; *p* = 0.009), diabetes (79% vs 64.6%; *p* = 0.047) and hypertension (88.3% vs. 67%; *p* = 0.002) (Table 2).

**Table 2:** Characteristics of elderly groups with advanced fibrosis (F3–4) vs. non-advanced fibrosis (F0–2)

Elderly NAFLD	Non-advanced fibrosis	Advanced fibrosis	P-value
Characteristics, median (IQR); n (%)	(***n*** = 113)	(***n*** = 62)	
**Age (year)**	64 (62–66)	65 (62–68)	0.118
**Female**	57 (50.4%)	45 (72.6%)	**0.005**
**Weight (kg)**	68.35 (59.88–78.00)	70.7 (64–79)	0.565
**Height (m)**	1.6 (1.57–1.70)	1.6 (1.54–1.68)	0.227
**BMI (kg/m**^**2**^**)**	26.68 (23.94–29.12)	27.55 (25.89–31.22)	0.055
**BMI ≥ 25 (kg/m**^**2**^)	74 (65.5%)	52 (83.9%)	**0.01**
**Waist circumference (cm)**	92.05 (84–100)	96 (89.50–102.50)	**0.017**
**Hip circumference (cm)**	98 (92.00–104.63)	101 (95.50–109)	0.158
**Central obesity**	72 (79.1%)	54 (94.7%)	**0.009**
**Diabetes**	73 (64.6%)	49 (79.0%)	**0.047**
**Hypertension**	69 (67%)	53 (88.3%)	**0.002**
**Dyslipidemia**	94 (83.2%)	54 (87.1%)	0.493
**Albumin (g/L)**	43 (42–45)	43 (40–46)	0.637
**ALT (U/L)**	52.5 (37–89.75)	66 (36–91)	0.276
**AST (U/L)**	38.5 (27.25–56.75)	51 (34–82)	0.086
**ALP (U/L)**	63 (49–117.25)	69.5 (69–70)	0.615
**GGT (U/L)**	48 (34.25–70.75)	75 (42.75–122.5)	**0.002**
**HbA1c (%)**	6.5 (5.9–7.4)	6.7 (6.13–7.28)	0.435
**Total cholesterol (mmol/L)**	4.4 (3.5–5.38)	4.35 (3.53–5.1)	0.483
**LDL cholesterol (mmol/L)**	2.32 (1.8–3.23)	2.23 (1.7–3.08)	0.389
**HDL cholesterol (mmol/L)**	1.2 (1.03–1.4)	1.3 (1.1–1.5)	0.068
**Triglycerides (mmol/L)**	1.4 (1.2–1.79)	1.5 (1.13–1.8)	0.833
**Hemoglobin (g/dL)**	14.1 (13.4–14.8)	13.45 (12.63–14.68)	0.055
**Platelet (10**^**9**^**/L)**	229.5 (193–284.25)	205 (145.75–243.25)	**0.003**
**WBC (10**^**9**^**/L)**	7 (5.8–8.23)	6.9 (5.83–8.15)	0.638
**NAFLD Activity Score (NAS)**	4 (3–5)	4 (4–5)	**0.023**
**NAS ≥ 5**	32 (28.3%)	26 (41.9%)	0.067
**Platelet count** **<** **140 × 10**^**9**^**/L**	10 (8.8%)	13 (21%)	**0.023**
**NAFL fibrosis score (NFS)**^a^	−1.00 (1.48)	−0.24 (1.4)	**0.005**
**APRI score**^a^	0.91 (1.41)	1.74 (2.85)	0.077
**FIB-4 score**^a^	3.06 (5.15)	5.57 (9.01)	0.095

*APRI* AST to platelet ratio index, *BMI* body mass index; Non-Alcoholic Steatohepatitis (NAS) score

^a^mean (SD)

By univariate analysis, female sex, obesity, central obesity, diabetes and hypertension were associated with advanced fibrosis among the elderly NAFLD patients. By multivariable analysis, factors associated with advanced fibrosis in elderly patients included female sex (OR 3.21; 95%CI 1.37–7.54) and hypertension (OR 3.68; 95%CI 1.11–12.23). In contrast, diabetes (OR 3.38; 95%CI 1.70–6.73) and platelet count less than 140 × 10^9^/L (OR 4.18; 95%CI 1.32–13.25) were factors associated with advanced fibrosis in non-elderly patients (Table 3).

**Table 3:** Univariate analysis and multivariate analysis for predictors of advanced fibrosis in the elderly and non-elderly patients with NAFLD

Characteristics	Elderly NAFLD patients				Non-elderly NAFLD patients			
	Univariate analysis		Multivariate analysis		Univariate analysis		Multivariate analysis	
	OR (95%CI)	P-value	OR (95%CI)	P-value	OR (95%CI)	P-value	OR (95%CI)	P-value
**Age** **>** **70 years**	2.22 (0.85–5.80)	0.103	2.24 (0.43–11.61)	0.338				
**Female**	2.60 (1.33–5.08)	**0.005**	**3.21 (1.37–7.54)**	**0.007**	0.89 (0.53–1.47)	0.639	0.59 (0.3–1.15)	0.119
**BMI** **>** **25 kg/m**^**2**^	2.74 (1.25–6.00)	**0.011**	2.41 (0.85–6.8)	0.098	1.13 (0.55–2.31)	0.741	0.94 (0.35–2.54)	0.904
**Central obesity**	4.75 (1.34–16.88)	**0.016**	4.09 (0.68–24.67)	0.124	1.24 (0.53–2.88)	0.62	1.18 (0.44–3.18)	0.747
**Diabetes mellitus**	2.07 (1.00–4.26)	**0.049**	2.29 (0.88–5.93)	0.088	3.88 (2.22–6.79)	**< 0.001**	3.38 (1.7–6.73)	**0.001**
**Hypertension**	3.73 (1.53–9.07)	**0.004**	**3.68 (1.11–12.23)**	**0.034**	1.29 (0.76–2.21)	0.346	1.59 (0.8–3.12	0.183
**Dyslipidemia**	1.36 (0.56–3.33)	0.494			1.01 (0.58–1.76)	0.97		
**Platelet count** **<** **140 × 10**^**9**^**/L**	2.73 (1.12–6.67)	**0.027**	2.43 (0.59–9.91)	0.217	4.06 (1.85–8.88)	**< 0.001**	4.18 (1.32–13.25)	**0.015**

*OR* odd ratios, *CI* confidence intervals

### The performance of non-invasive fibrosis scores in elderly NAFLD patients

**Fig. 2:**
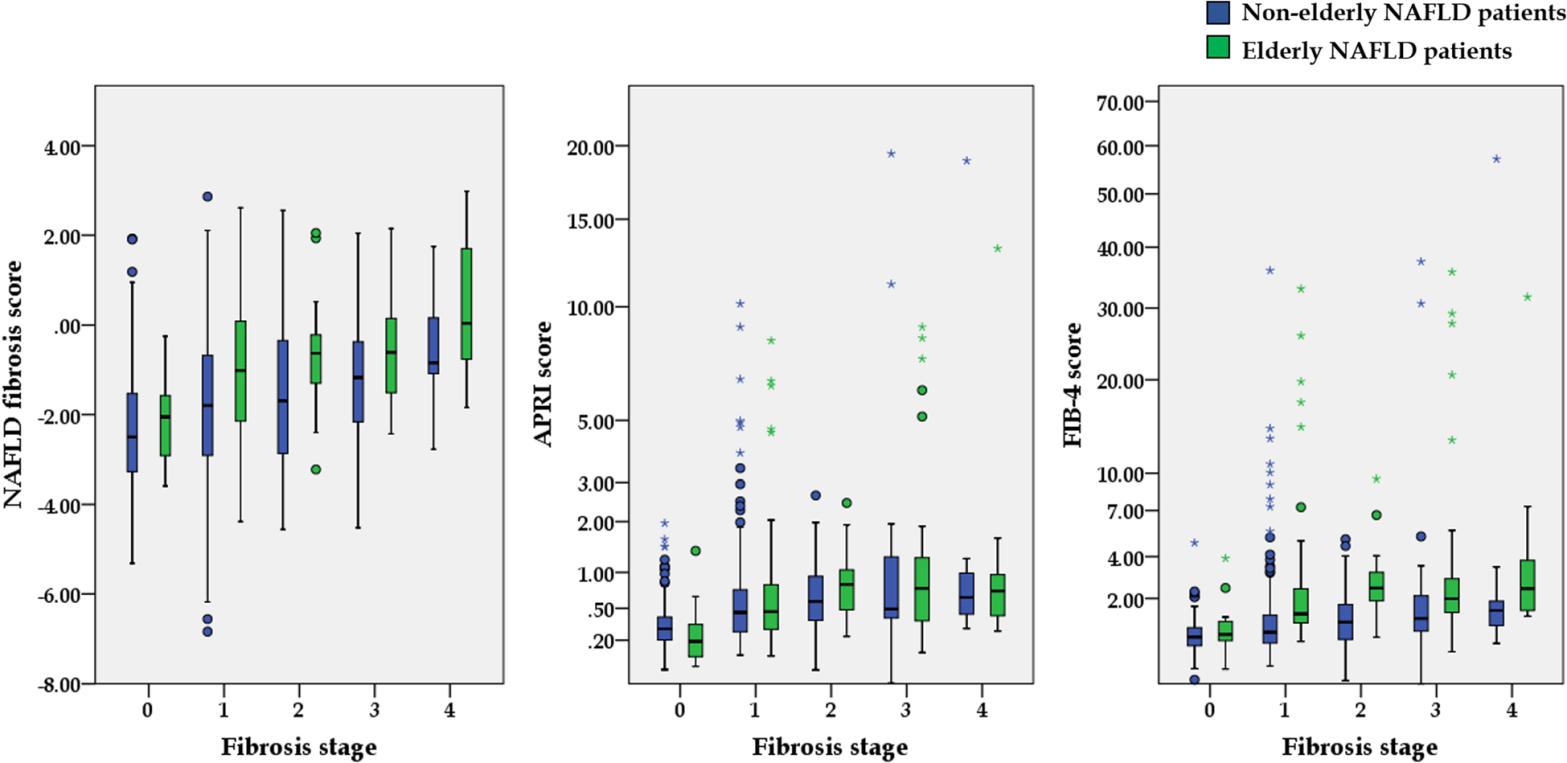
Box plot of non-invasive fibrosis scores in each liver biopsy-proven fibrosis stage

Box plot of non-invasive fibrosis scores in each liver biopsy-proven fibrosis stage is shown in Fig. 2.The AUROCs of APRI, NFS and FIB-4 to predict advanced fibrosis in the elderly NAFLD patients were as follows: 0.62 (0.52–0.72) *p* = 0.03, 0.65 (0.55–0.75) *p* = 0.01, and 0.64 (0.54–0.74) p = 0.01, respectively (Table 4). Comparison of the AUROCs among APRI, NFS, and FIB-4 showed no significant difference within elderly NAFLD patients (APRI vs NFS, *p* = 0.551, APRI vs FIB-4, *p* = 0.308 and NFS vs FIB-4, *p* = 0.828). The flow diagram evaluating the performance of non-invasive fibrosis scores in elderly NAFLD patients is shown in supplemental fig. 1. Comparison between percentage of advanced fibrosis in elderly NAFLD patients and non-elderly NAFLD patients from each non-invasive fibrosis score is shown in supplemental table 3. Sensitivity, specificity, positive predictive value (PPV) and negative predictive value (NPV) of APRI, NFS and FIB-4 in both elderly and non-elderly NAFLD are shown in supplemental table 4.

**Table 4:** The performance of AST to Platelet Ratio Index (APRI), NAFLD fibrosis score (NFS) and fibrosis 4 (FIB-4) in prediction of advanced fibrosis in elderly NAFLD patients and non-elderly NAFLD patients

Area under the curve (95%CI)	APRI	NFS	FIB-4
Non-elderly NAFLD	0.67 (0.60–0.74) p < 0.001	0.68 (0.61–0.75) p < 0.001	0.71 (0.64–0.78) p < 0.001
	(*n* = 480)	(*n* = 475)	(n = 480)
Elderly NAFLD	0.62 (0.52–0.72) p = 0.03	0.65 (0.55–0.75) p = 0.01	0.64 (0.54–0.74) p = 0.01
	(*n* = 132)	(*n* = 131)	(n = 132)
All patients	0.66 (0.61–0.72) p < 0.001	0.70 (0.65–0.75) *p* < 0.001	0.73 (0.67–0.78) p < 0.001
	(*n* = 612)	(*n* = 606)	(n = 612)

## Discussion

This multicenter study on 700 biopsy-proven NAFLD patients revealed that a significant proportion of elderly patients had advanced fibrosis (35.4%). Liver is an organ with age and sex-related changes [24], and the complications from chronic liver disease increase with age, contributing significantly to health care burden. Besides, we demonstrated that the non-invasive scores were higher in elderly NAFLD patients than non-elderly patients. To better understand how to hinder disease progression, reliable baseline data are needed to identify predictors for early intervention and close monitoring. In this study, we found that elderly NAFLD patients with advanced fibrosis were more likely to be female and were more likely to have hypertension.

An earlier study from Noureddin et al. compared between elderly and non-elderly patients with NAFLD showed the predominance of NAFLD in elderly female patients. Their elderly NAFLD patients were more likely to be hypertensive, had lower mean BMI and smaller waist circumference. Moreover, They had a higher mean AST/ALT ratio, lower mean platelet count, and higher mean APRI score that were suggested as indices of more advanced liver disease [3]. Similar to the previous study, our elderly NAFLD patients had lesser mean BMI and hip circumference. This group of patients was more likely to have hypertension, diabetes mellitus, and dyslipidemia. It is worth noting that we must be aware that the elderly group may have NAFLD even if they have low BMI.

To identify patients who have advanced fibrosis, liver biopsy is the gold standard, but this procedure has some limitations due to its invasive nature and the potential complications. Non-invasive fibrosis scores, for example, APRI, NFS and FIB-4 score were developed to predict liver fibrosis. However, the validation of these scores were included only in the minority of elderly group [25] or some studies did not include any subjects with ages ≥65 years [26, 27]. Meta-analysis in 2016, most of patients aged less than 60, showed FIB-4 score had better diagnostic accuracy than NFS and BARD score. In non-elderly NAFLD, our study also supported that FIB-4 had better diagnostic accuracy than NFS and APRI [28].

Recently, McPherson et al. reported that the NAFLD fibrosis score and FIB-4 had low specificity for advanced fibrosis in patients aged ≥65 years leading to a high false positive rate. New cutoffs have been proposed to improve the accuracy of the NFS and FIB-4 score in patients ages ≥65 years [17]**.** From our data, we found that the NFS and FIB-4 had a lower specificity when using lower cutoffs. When validating by using the new cutoffs by McPherson et al., specificity was increased in elderly, however, sensitivity was low (supplemental table 5).

Aging promotes metabolic disturbances causing an increase in lipid accumulation in non-adipose tissues, including the liver. With reduced physical activity in aging, insulin resistance and hyperinsulinemia promote obesity and the onset of the metabolic syndrome. NASH is considered to be a manifestation of metabolic syndrome [29, 30]. In the present study, the prevalence of advanced liver fibrosis was high in elderly patients, particularly in women with central obesity. The gender difference of the metabolic disturbance is most evident in postmenopausal women. Although the mechanisms of menopause-induced metabolic dysfunction are largely unknown, estrogen depletion is thought to responsible for the underlying pathological mechanism [31]. This observation requires further study to confirm.

The strength of this study includes the use of a well-characterized cohort of biopsy-proven NAFLD patients from multiple centers in Asia. The proportion of elderly patients in the present study is greater than those reported in previous studies [3, 4]. One of the limitations may arise from the liver biopsy results and its assessment which was reported by the local pathologists of each center and they were not reviewed centrally by a panel of pathologists. However, interobserver agreement for advanced fibrosis tends to be better, and our study focused on advanced fibrosis. Secondly, the sampling errors of liver biopsy and observer variability may affect the pathological results interpretation [22]. Lastly, the study could be affected by selection bias as the sample came from tertiary centers and specialist liver clinics, thus the findings may not be applicable to general clinic setting.

## Conclusions

Elderly patients with NAFLD had a higher prevalence of advanced fibrosis than non-elderly patients. Female sex and hypertension were predictive factors for the presence of advanced fibrosis in elderly. We should be aware of this in our aging society. Performance of fibrosis prediction by using non-invasive scoring system including APRI, NFS and FIB-4 were not good enough to predict the presence of advanced fibrosis stage in the elderly.

## Supplementary information

**Additional file 1: Supplemental fig. 1.** The flow diagram evaluating the performance of non-invasive fibrosis scores in elderly and non-elderly NAFLD patients. **Supplemental table 1.** The percentages of elderly NAFLD patients (*n* = 175) and non-elderly NAFLD patients from each country included in the study (total = 1008). **Supplemental table 2.** Characteristics of elderly and non-elderly NAFLD patients in different centers. **Supplemental table 3.** Comparison between percentages of advanced fibrosis in elderly and non-elderly NAFLD patients. **Supplemental table 4.** Thesensitivity, specificity, positive predictive value (PPV) and negative predictive value (NPV) of APRI, NFS and FIB-4 in both elderly and non-elderly NAFLD patients. **Supplemental table 5.** AUROC, sensitivity, specificity, positive predictive value (PPV) and negative predictive value (NPV) of APRI, NFS and FIB-4 in elderly NAFLD patients when elderly defined as ≥ 65 according to McPherson et al.

## Data Availability

The datasets used and/or analyzed during the current study are available from the corresponding author on reasonable request.

## Abbreviations

APRI: Aspartate aminotransferase-to-platelet ratio index
AUROC: Area under receiver-operating characteristics curve
FIB-4: Fibrosis 4 score
NAFLD: Nonalcoholic fatty liver disease
NAS: NAFLD activity score
NFS: NAFLD fibrosis score
NPV: Negative predictive value
PPV: Positive predictive value
