# Supplementary material for: Predictors of advanced fibrosis in elderly patients with biopsy-confirmed nonalcoholic fatty liver disease: the GOASIA study
Source: BMC Gastroenterol. 2020 Apr 6;20:88. doi: 10.1186/s12876-020-01240-z (PMC7137333; doi:10.1186/s12876-020-01240-z)
Supplement: Supplementary file 1 — Additional file 1: Supplemental fig. 1. The flow diagram evaluating the performance of non-invasive fibrosis scores in elderly and non-elderly NAFLD patients. Supplemental table 1. The percentages of elderly NAFLD patients (n = 175) and non-elderly NAFLD patients from each country included in the study (total = 1008). Supplemental table 2. Characteristics of elderly and non-elderly NAFLD patients in different centers. Supplemental table 3. Comparison between percentages of advanced fibrosis in elderly and non-elderly NAFLD patients. Supplemental table 4. Thesensitivity, specificity, positive predictive value (PPV) and negative predictive value (NPV) of APRI, NFS and FIB-4 in both elderly and non-elderly NAFLD patients. Supplemental table 5. AUROC, sensitivity, specificity, positive predictive value (PPV) and negative predictive value (NPV) of APRI, NFS and FIB-4 in elderly NAFLD patients when elderly defined as ≥ 65 according to McPherson et al. [file 12876_2020_1240_MOESM1_ESM.docx]

**Supplementary material**

**Predictors of advanced fibrosis in elderly patients with biopsy-confirmed nonalcoholic fatty liver disease: The GOASIA study**

Panyavee Pitisuttithum1, Wah Kheong Chan2, Panida Piyachaturawat1, Kento Imajo3, Atsushi Nakajima3, Yosuke Seki4, Kazunori Kasama4, Satoru Kakizaki5, Jian Gao Fan6, Myeong Jun Song7, Seung Kew Yoon7, Yock Young Dan8, Laurentius Lesmana9, Khek Yu Ho8, Khean Lee Goh2, Vincent Wai Sun Wong10, Sombat Treeprasertsuk1

1Department of Medicine, Chulalongkorn University, Bangkok, Thailand

2Department of Medicine, University of Malaya, Kuala Lumpur, Malaysia

3Department of Gastroenterology and Hepatology, Yokohama City University Graduate School of Medicine, Yokohama, Japan.4Weight Loss and Metabolic Surgery Centre, Yotsuya Medical Cube, Tokyo,

5 Department of Medicine and Molecular Science, Gunma University Graduate School of Medicine, Gunma, Japan,

6 Department of Gastroenterology, Shanghai Jiaotong University School of Medicine, Shanghai, China,

7Department of Internal Medicine, The Catholic University Korea, Seoul, Korea

8Department of Medicine, National University of Singapore, Singapore

9Digestive Disease and Oncology GI Centre, Medistra Hospital, Jakarta, Indonesia,

10 Department of Medicine and Therapeutics, the Chinese University of Hong Kong, Hong Kong, China.

**Supplemental figure1** the flow diagram evaluating the performance of non-invasive fibrosis scores in elderly NAFLD patients and non-elderly NAFLD patients.

**
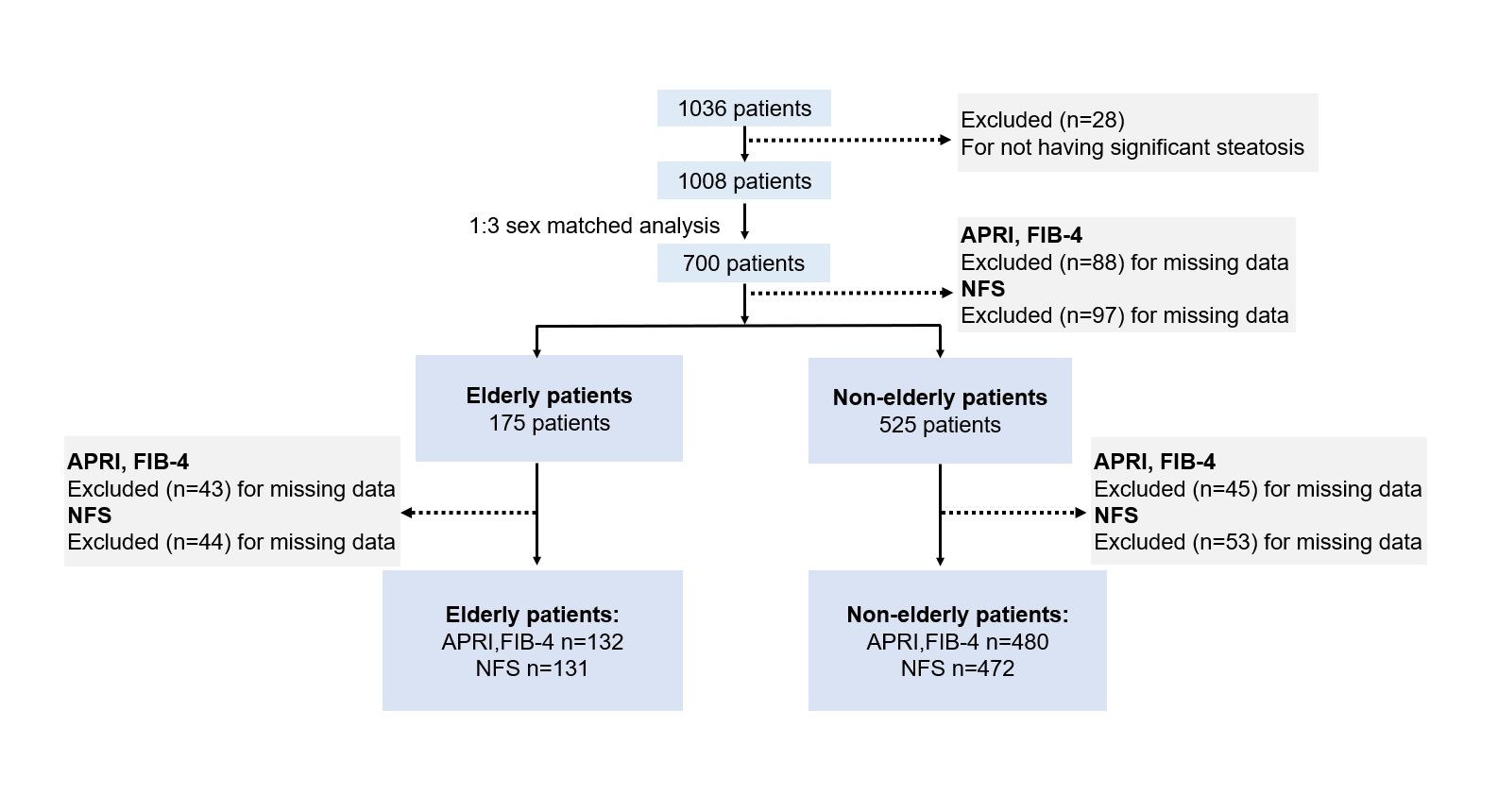
**

**Supplemental table1** showed the percentage of elderly NAFLD patients (n=175) and non-elderly NAFLD patients from each country included in the study (total=1008).


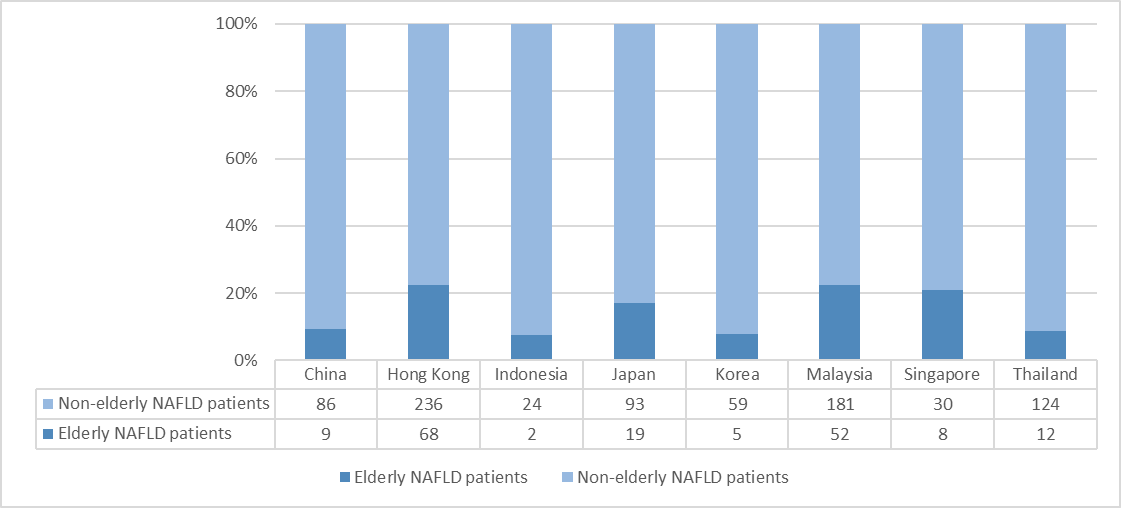


**Supplemental table2** showed characteristics of elderly and non-elderly NAFLD patients in different centers.

| **Centers** | **Hong Kong** | | | **Malaysia** | | | **Thailand** | | | **Japan** | | |
| --- | --- | --- | --- | --- | --- | --- | --- | --- | --- | --- | --- | --- |
| **Characteristics** | **Non-elderly** | **Elderly** | **P valve** | **Non-elderly** | **Elderly** | **P valve** | **Non-elderly** | **Elderly** | **P valve** | **Non-elderly** | **Elderly** | **P valve** |
| **Age (year)** | 47.5 | 66.1 | **0.00** | 47.8 | 63.8 | **0.00** | 38.7 | 65.6 | **0.00** | 42.6 | 64.4 | **0.00** |
| **Female** | 61 (42.4%) | 33 (48.5%) | 0.40 | 38 (35.5%) | 17 (32.7%) | 0.73 | 32 (34.4%) | 6 (36.2%) | 0.29 | 26 (42.6%) | 8 (42.1%) | 0.99 |
| **Weight (kg)** | 77.3 | 72.4 | **0.02** | 77.4 | 69.5 | **0.00** | 113.7 | 69.4 | **0.00** | 112.9 | 81.3 | **0.00** |
| **Height (m)** | 1.6 | 1.6 | 0.16 | 1.6 | 1.6 | 0.82 | 1.6 | 1.6 | **0.06** | 1.7 | 1.6 | **0.00** |
| **BMI (kg/m^2^)** | 28.5 | 27.8 | 0.23 | 30.5 | 27.5 | **0.00** | 41.5 | 27.8 | **0.00** | 40.7 | 32.3 | **0.00** |
| **Waist circumference (cm)** | 92.7 | 98.5 | **0.04** | 97.6 | 94.5 | 0.06 | 103.9 | 94.0 | **0.03** | 101.9 | 98.7 | 0.17 |
| **Hip circumference (cm)** | 100.4 | 97.2 | 0.21 | 107.6 | 103.5 | **0.01** | 111.8 | 105.6 | 0.23 | n/a | n/a |  |
| **Diabetes** | 75 (52.1%) | 59 (86.8%) | **0.00** | 52 (48.6%) | 36 (69.2%) | **0.01** | 51 (54.8%) | 10 (83.3%) | 0.06 | 30 (49.2%) | 12 (63.25) | 0.29 |
| **Hypertension** | 64 (44.4%) | 55 (80.9%) | **0.00** | 58 (54.2%) | 43 (82.7%) | **0.00** | n/a | n/a |  | 38 (62.3%) | 15 (78.9%) | 0.18 |
| **Albumin (g/L)** | 44.8 | 44.1 | 0.10 | 42.9 | 42.1 | 0.22 | 43.8 | 43.2 | 0.49 | 41.9 | 43.7 | 0.06 |
| **ALT (U/L)** | 67.8 | 47.7 | **0.00** | 76.8 | 67.8 | 0.23 | 63.3 | 97.3 | **0.01** | 67.4 | 59.5 | 0.62 |
| **AST (U/L)** | 39.1 | 32.5 | 0.40 | 48.4 | 51.7 | 0.52 | 63.3 | 97.3 | 0.01 | 45.1 | 44.9 | 0.98 |
| **ALP (U/L)** | n/a | n/a |  | n/a | n/a |  | n/a | n/a |  | n/a | n/a |  |
| **GGT (U/L)** | 76.8 | 78.7 | 0.89 | 85.8 | 104.4 | 0.22 | 46.7 | 54.3 | 0.52 | 59.1 | 71.2 | 0.34 |
| **HbA1c (%)** | 6.4 | 7.0 | **0.00** | 6.6 | 6.9 | 0.32 | n/a | n/a |  | 6.4 | 6.4 | 0.93 |
| **Total cholesterol (mmol/L)** | 5.0 | 4.3 | **0.00** | 5.0 | 4.6 | **0.03** | n/a | n/a |  | 5.2 | 5.5 | 0.33 |
| **LDL cholesterol (mmol/L)** | 3.0 | 2.3 | **0.00** | 3.1 | 2.6 | **0.01** | n/a | n/a |  | 3.6 | 3.4 | 0.66 |
| **HDL cholesterol (mmol/L)** | 1.5 | 1.4 | 0.14 | 1.2 | 1.2 | 0.33 | n/a | n/a |  | 1.2 | 1.5 | 0.02 |
| **Triglycerides (mmol/L)** | 1.7 | 1.5 | 0.28 | 1.7 | 1.7 | 0.82 | n/a | n/a |  | 1.6 | 1.3 | 0.33 |
| **Hemoglobin (g/dL)** | 14.0 | 13.7 | 0.07 | 13.9 | 14.2 | 0.27 | 13.5 | 13.8 | 0.45 | 14.1 | 14.1 | 0.95 |
| **Platelet (10^9^/L)** | 243.7 | 214.4 | **0.00** | 278.8 | 258.1 | **0.07** | 274.1 | 233.9 | 0.07 | 202.4 | 99.1 | 0.00 |
| **WBC (10^9^/L)** | 7.0 | 6.8 | 0.47 | 8.1 | 7.7 | 0.26 | 8.3 | 6.6 | 0.07 | 6.6 | 5.9 | 0.30 |
| **NAFLD Activity Score (NAS)** | 3.6 | 3.6 | 0.95 | 4.4 | 4.5 | 0.92 | 3.7 | 4.0 | 0.61 | 5.0 | 3.9 | 0.00 |
| **Advanced fibrosis (≥F3)** | 31 (21.5%) | 27 (39.70) | **0.01** | 11 (10.3%) | 21 (40.4%) | **0.00** | 7 (7.5%) | 2 (16.7%) | 0.29 | 3 (4.9%) | 6 (31.6%) | **0.00** |

| **Centers** | **China** | | | **Korea** | | | **Singapore** | | | **Indonesia** | | |
| --- | --- | --- | --- | --- | --- | --- | --- | --- | --- | --- | --- | --- |
| **Characteristics** | **Non-elderly** | **Elderly** | **P valve** | **Non-elderly** | **Elderly** | **P valve** | **Non-elderly** | **Elderly** | **P valve** | **Non-elderly** | **Elderly** | **P valve** |
| **Age (year)** | 38.9 | 62.4 | **0.00** | 41.0 | 63.0 | **0.00** | 45.4 | 66.3 | **0.00** | 41.9 | 60.5 | **0.02** |
| **Female** | 31 (63.3%) | 4 (44.4%) | 0.29 | 18 (51.4%) | 1 (20%) | 0.19 | 9 (42.9%) | 4 (50%) | 0.73 | 4 (44.8%) | 0.0 | 0.40 |
| **Weight (kg)** | 74.3 | 72.3 | 0.58 | 79.2 | 62.5 | **0.02** | 75.7 | 68.2 | 0.25 | 66.5 | 54.0 | 0.45 |
| **Height (m)** | 1.7 | 1.7 | 0.44 | 1.7 | 1.6 | **0.03** | 1.6 | 1.5 | 0.06 | 1.6 | 1.5 | **0.04** |
| **BMI (kg/m^2^)** | 27.0 | 25.5 | 0.19 | 28.7 | 25.5 | 0.05 | 28.9 | 28.9 | 0.99 | 25.6 | 23.7 | 0.73 |
| **Waist circumference (cm)** | 60.6 | 67.1 | 0.80 | 18.5 | 36.3 | 0.66 | n/a | n/a |  | n/a | n/a |  |
| **Hip circumference (cm)** | 66.4 | 75.4 | 0.74 | 16.8 | 51.4 | 0.44 | n/a | n/a |  | n/a | n/a |  |
| **Diabetes** | 10 (20.4%) | 1 (11.1%) | 0.51 | 7 (20%) | 0.00 | 0.27 | 10 (47.6%) | 3 (37.5%) | 0.62 | 2 (13.3%) | 1 (50%) | 0.20 |
| **Hypertension** | 7 (14.3%) | 5 (55.6%) | **0.01** | 16 (45.7%) | 3 (60%) | 0.55 | 5 (23.8%) | 1 (12.5%) | 0.50 | 1 (6.7%) | 0.0 | 0.71 |
| **Albumin (g/L)** | 44.5 | 39.8 | **0.01** | 44.5 | 44.5 | 0.99 | 43.4 | 44.6 | 0.59 | 42.7 | 41.0 | 0.61 |
| **ALT (U/L)** | 55.7 | 30.9 | **0.03** | 113.3 | 72.6 | **0.01** | 84.0 | 92.1 | 0.67 | 85.7 | 12.5 | 0.31 |
| **AST (U/L)** | 46.2 | 53.1 | 0.56 | 64.8 | 48.2 | 0.34 | 79.5 | 114.0 | 0.15 | 65.7 | 12.0 | 0.28 |
| **ALP (U/L)** | n/a | n/a |  | n/a | n/a |  | 71.5 | 78.8 | 0.59 | n/a | n/a |  |
| **GGT (U/L)** | 110.9 | 95.4 | 0.85 | 87.1 | 39.5 | 0.32 | n/a | n/a |  | 101.5 | 45.5 | 0.73 |
| **HbA1c (%)** | n/a | n/a |  | 7.3 | n/a |  | 7.3 | 7.1 | 0.70 | 6.8 | . |  |
| **Total cholesterol (mmol/L)** | 5.3 | 4.9 | 0.38 | 5.2 | 4.6 | 0.19 | 5.3 | 5.6 | 0.59 | 5.7 | 5.6 | 0.97 |
| **LDL cholesterol (mmol/L)** | 3.3 | 3.1 | 0.61 | 3.2 | 2.8 | 0.24 | 3.1 | 3.4 | 0.53 | 2.8 | 3.7 | 0.29 |
| **HDL cholesterol (mmol/L)** | 1.4 | 1.4 | 0.76 | 1.1 | 1.2 | 0.53 | 1.2 | 1.2 | 0.90 | 1.1 | 1.5 | 0.26 |
| **Triglycerides (mmol/L)** | 2.1 | 1.7 | 0.45 | 2.1 | 1.5 | 0.01 | 2.4 | 2.3 | 0.97 | 1.5 | 1.7 | 0.76 |
| **Hemoglobin (g/dL)** | 14.5 | 13.8 | 0.22 | 14.8 | 15.1 | 0.67 | 14.0 | 14.5 | 0.49 | 13.4 | 11.5 | 0.16 |
| **Platelet (10^9^/L)** | 216.3 | 197.1 | 0.40 | 243.0 | 229.0 | 0.62 | 262.2 | 230.5 | 0.35 | 241.9 | 352.5 | **0.03** |
| **WBC (10^9^/L)** | 6.6 | 6.4 | 0.72 | 6.7 | 6.0 | 0.25 | 8.1 | 7.1 | 0.48 | 6.6 | 7.6 | 0.53 |
| **NAFLD Activity Score (NAS)** | 4.1 | 4.2 | 0.81 | 4.6 | 4.0 | 0.41 | 4.8 | 5.3 | 0.37 | 3.9 | 2.0 | 0.14 |
| **Advanced fibrosis (≥F3)** | 7 (14.3%) | 3 (33.3%) | 0.16 | 4 (11.4%) | 1 (20%) | 0.59 | 6 (28.6%) | 2 (25%) | 0.85 | 1 (6.7%) | 0.0 | 0.81 |

**Supplemental table3** showed comparison between percentage of advanced fibrosis in elderly NAFLD patients and non-elderly NAFLD patients.

| Patient with advanced fibrosis | All patients | Elderly NAFLD patients | Non-elderly NAFLD patients |
| --- | --- | --- | --- |
| Non-invasive fibrosis scores | | | |
| APRI | 105 (15%)  (n=612) | 33 (18.9%)  (n=132) | 72 (13.7%)  (n=480) |
| FIB-4 | 70 (10%)  (n=612) | 36 (20.6%)  (n=132) | 34 (6.5%)  (n=480) |
| NFS | 5.3 (7.6%)  (n=603) | 23 (13.1%)  (n=131) | 30 (5.7%)  (n=472) |
| Biopsy proven  advanced fibrosis | 132 (18.5%)  (n=700) | 62 (35.4%)  (n=175) | 70 (13.4%)  (n=525) |

**Supplemental table4** showed sensitivity, specificity, positive predictive value (PPV) and negative predictive value (NPV) of APRI, NFS and FIB-4 in both elderly and non-elderly NAFLD.

| Test | Cut-offs | Sensitivity | Specificity | PPV | NPV |
| --- | --- | --- | --- | --- | --- |
| Elderly NAFLD | | | | | |
| APRI | ≥1 | 30.2% | 77.5% | 39.4% | 69.7% |
| NFS | >0.676 | 25.6% | 86.4% | 47.8% | 70.4% |
|  | >0.12^a^ | 30.2% | 78.4% | 40.6% | 69.7% |
|  | >-1.455 | 74.4% | 40.9% | 38.1% | 76.6% |
| FIB-4 | >2.67 | 32.6% | 75.3% | 38.9% | 69.8% |
|  | >2^a^ | 51.2% | 67.4% | 43.1% | 74.1% |
|  | >1.3 | 88.4% | 38.2% | 40.9% | 87.2% |
| Non-elderly NAFLD | | | | | |
| APRI | ≥1 | 29.8% | 87.0% | 23.6% | 90.2% |
| NFS | >0.676 | 10.5% | 94.2% | 20.0% | 88.5% |
|  | >-1.455 | 66.7% | 64.8% | 20.7% | 93.4% |
| FIB-4 | >2.67 | 12.3% | 93.6% | 20.6% | 88.8% |
|  | >1.3 | 54.4% | 75.7% | 23.1% | 92.5% |

**NOTE:** ^a^ cut off according to McPherson et al (18).

**Supplemental table 5** showed AUROC, sensitivity, specificity, positive predictive value (PPV) and negative predictive value (NPV) of APRI, NFS and FIB-4 in elderly NAFLD when elderly defined as≥65 according to McPherson et al.

| Elderly NAFLD (defined as age≥65) | | | | | | | |
| --- | --- | --- | --- | --- | --- | --- | --- |
| Test | **AUROC** | **P-valve** | **Cut-offs** | **Sensitivity** | **Specificity** | **PPV** | **NPV** |
| APRI | 0.57 (0.41-0.73) | 0.42 | ≥1 | 26.3% | 73.0% | 33.3% | 65.9% |
| NFS | 0.73 (0.59-0.87) | 0.01 | >0.676 | 31.6% | 86.5% | 54.5% | 71.1% |
|  |  |  | >0.12^a^ | 36.8% | 78.4% | 46.7% | 70.7% |
|  |  |  | >-1.455 | 89.5% | 32.4% | 40.5% | 85.7% |
| FIB-4 | 0.61 (0.46-0.77) | 0.17 | >2.67 | 42.1% | 70.3% | 42.1% | 70.3% |
|  |  |  | >2^a^ | 57.9% | 62.2% | 44.0% | 74.2% |
|  |  |  | >1.3 | 78.9% | 37.8% | 39.5% | 77.8% |

**NOTE:** a cut off according to McPherson et al
